# Allergic fungal airways disease (AFAD): an under-recognised asthma endotype

**DOI:** 10.1007/s11046-021-00562-0

**Published:** 2021-05-27

**Authors:** Catherine H. Pashley, Andrew J. Wardlaw

**Affiliations:** Department of Respiratory Sciences, Institute for Lung Health, University of Leicester, University Road, Leicester, LE1 7RH UK

**Keywords:** ABPA, SAFS, AFAD, Thermotolerant fungi, Fungal sensitisation

## Abstract

The term allergic fungal airways disease has a liberal definition based on IgE sensitisation to thermotolerant fungi and evidence of fungal-related lung damage. It arose from a body of work looking into the role of fungi in asthma. Historically fungi were considered a rare complication of asthma, exemplified by allergic bronchopulmonary aspergillosis; however, there is a significant proportion of individuals with *Aspergillus fumigatus* sensitisation who do not meet these criteria, who are at high risk for the development of lung damage. The fungi that play a role in asthma can be divided into two groups; those that can grow at body temperature referred to as thermotolerant, which are capable of both infection and allergy, and those that cannot but can still act as allergens in IgE sensitised individuals. Sensitisation to thermotolerant filamentous fungi (*Aspergillus* and *Penicillium*), and not non-thermotolerant fungi (*Alternaria* and *Cladosporium*) is associated with lower lung function and radiological abnormalities (bronchiectasis, tree-in-bud, fleeting shadows, collapse/consolidation and fibrosis). For antifungals to play a role in treatment, the focus should be on fungi capable of growing in the airways thereby causing a persistent chronic allergenic stimulus and releasing tissue damaging proteases and other enzymes which may disrupt the airway epithelial barrier and cause mucosal damage and airway remodelling. All patients with IgE sensitisation to thermotolerant fungi in the context of asthma and other airway disease are at risk of progressive lung damage, and as such should be monitored closely.

## Introduction

Asthma is a common, global condition, affecting more than 300 million people and causing considerable morbidity in adults and children [1]. Asthma is characterised by airflow obstruction and chronic airway inflammation but is heterogeneous in its presentation. Traditionally, this variability in presentation has been described using single dimension, observable characteristics (phenotypes) based on triggers or patterns of symptoms such as exercise-induced or smoking-related asthma [2]. However, the greater focus in recent years on more difficult to control disease, where variability in presentation is greater than mild asthma, combined with a deeper understanding of disease processes, exposed this approach as too simplistic and not helpful in determining response to treatment or prognosis. A more multidimensional approach was advocated using complex biostatistical techniques to analyse data from different components of the disease process [3, 4]. This was applied to large disease cohorts enriched for difficult to control disease and the term endotypes applied to consistent patterns of asthma that transmitted coherently through different dimensions of the disease [2]. One of these endotypes was identified as a pattern of disease caused by fungal allergy (as measured by IgE sensitisation) to airway colonising, thermotolerant filamentous fungi with *Aspergillus fumigatus* as the archetypal mould involved, referred to as allergic bronchopulmonary mycosis (ABPM) [5], which we consider part of allergic fungal airway disease (AFAD), the focus of this review. An important aspect of IgE sensitisation to *A. fumigatus* and related fungi is that it is much more common in severe asthma, and where it does occur in mild asthma it is often due to cross-reactivity with skin colonising fungi such as *Malassezia* spp. which are not important in airway disease. Severe asthma is a difficult condition to define and is usually based on the amount of treatment prescribed which is partially dependent on factors other than underlying severity of disease [6]. One way to approach asthma severity is to deconstruct the degree of organ dysfunction into its component pathophysiology, an approach that reveals the connection between asthma and other airway diseases [7, 8]. This approach is also helpful in management of difficult to control disease, which is perhaps the main purpose of defining endotypes of asthma [9]. When taking this approach it is clear that an important pathophysiological abnormality caused by AFAD is lung damage, with a combination of fixed airflow obstruction, bronchiectasis and lung fibrosis.

Fungi are one of the major kingdoms of life. The kingdom is highly diverse, including taxa from numerous ecological niches with varied life history strategies and morphologies. The number of currently accepted fungal species is over 120 thousand, with estimates of the true number of fungal species being between 2.2 and 3.8 million [10]. Fungi are capable of causing human disease by direct infection, toxicoses, or allergy, with infection and allergy being the most relevant to chronic respiratory diseases including asthma (the focus of this review), chronic obstructive pulmonary disease (COPD) [11–13] and bronchiectasis [14, 15]. The fungal Kingdom is divided into 8 to 10 major groups, known as phyla, [16, 17]. Fungal pathogens are known to have evolved independently and repeatedly throughout the Kingdom [18]; however, only a small proportion are implicated in playing a role in asthma and these are mostly from the Ascomycota, Basidiomycota and Mucormycota [19]. The Mucormycota were part of the Zygomycota, an obsolete term still used in the literature, but the phyla was found to be polyphyletic and the group of relevance to asthma are found within the Mucormycota [20]. The fungi that play a role in asthma can be divided into two groups: those that can grow at body temperature, referred to as thermotolerant, which are capable of both infection and allergy, and those that cannot but can still act as allergens in IgE sensitised individuals. It is the thermotolerant group of filamentous fungi that cause AFAD.

## Allergic fungal airways disease

**Fig. 1:**
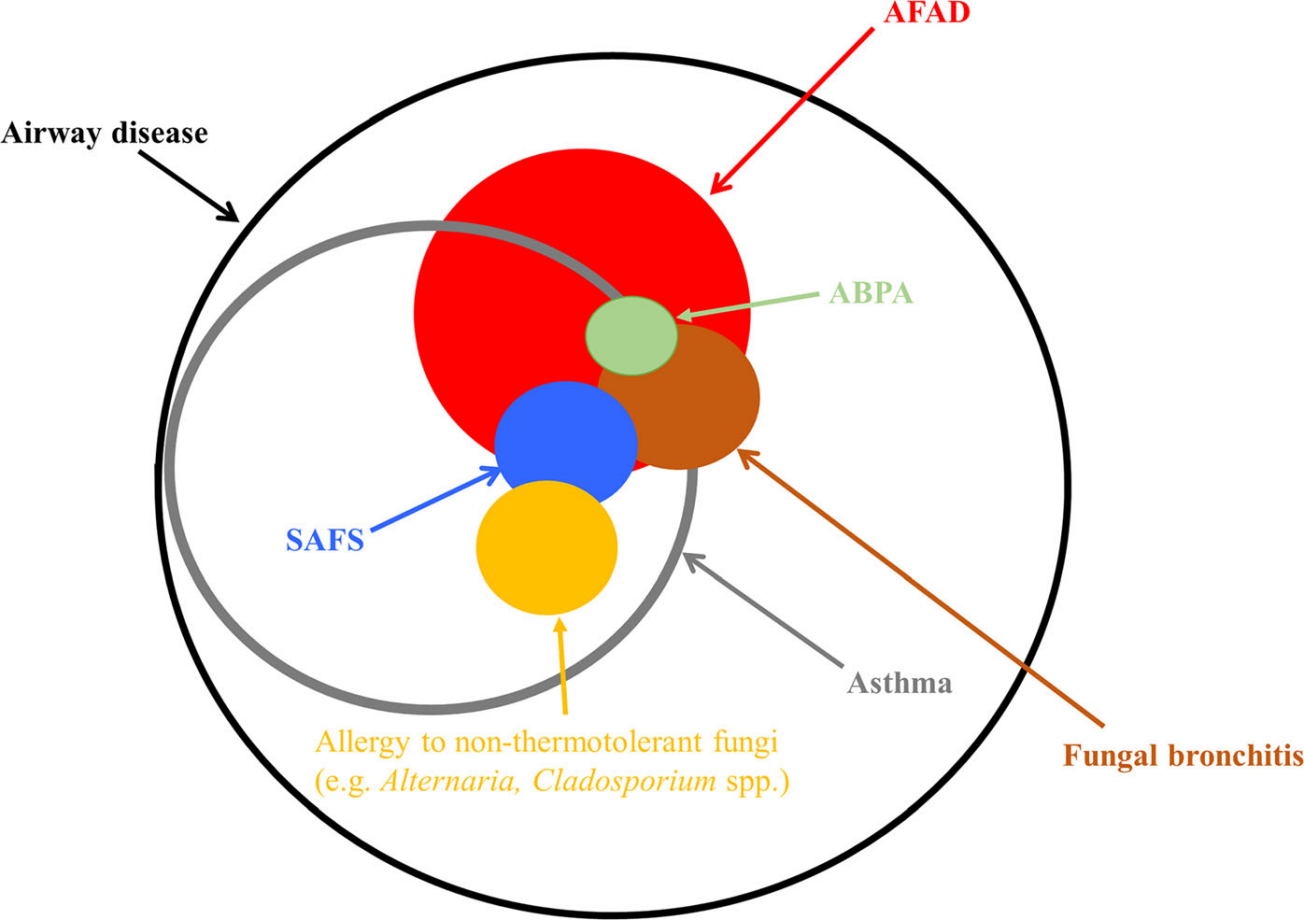
Venn diagram illustrating the relationship between various manifestations of airway disease complicated by fungal allergy, including allergic bronchopulmonary aspergillosis (ABPA), allergic fungal airways disease (AFAD) and severe asthma with fungal sensitisation (SAFS)

As noted above, the pathophysiological condition that is the subject of this review represents the host response to airway colonising, allergenic, thermotolerant, filamentous fungi, with *A. fumigatus* as the major culprit. The differing nomenclatures used to describe this process have caused confusion and prevented a clear understanding of the condition [21]. The initial descriptions of this endotype of airway disease were biased towards the clinically and immunologically florid presentation of what is a spectrum of disease. The term allergic bronchopulmonary aspergillosis/mycosis (ABPA/M) was coined using restrictive criteria developed from small cohorts of patients with limited statistical underpinning [22]. These criteria, if properly applied, excluded the great majority of people whose airway disease was complicated by allergy to *A. fumigatus* and related fungi. Not only was this true of asthma and cystic fibrosis, which were listed as predisposing conditions in the definition of ABPA, but it excluded presentations occurring in other airway diseases such as COPD and bronchiectasis, and sometimes de novo. This has caused uncertainty for clinicians in what the term ABPA actually represents and has meant that inclusion criteria for studies of fungal allergy have varied depending on the prejudices of the investigator. The criteria have since been modified a number of times, including recent attempts using larger cohorts and a statistical approach to defining biomarkers of disease [23–28]. The criteria proposed by the International Society for Human & Animal Mycology (ISHAM) are more relaxed making them more relevant to clinical practice [23], but still includes an arbitrary cut-off value for total IgE (> 1000 IU/L) which is not closely related to relevant clinical outcomes. Overall efforts at revision have suffered from flaws attendant on the lack of a gold standard for ABPA and the assumption that ABPA, as oppose to *A. fumigatus* sensitisation without ABPA, is a distinct clinical entity. As a result attempts to define ABPA have been largely tautologous in that the characteristics used to diagnose ABPA in the first place are then tested for their potential as criteria. Unbiased approaches to relating biomarkers to clinical outcomes such as the study discussed in detail below have revealed that only specific IgE sensitisation is reliably related to clinically relevant outcomes in asthma at least [29]. An alternative approach to solving the problem of ABPA only applying to a minority of patients was to create a new subgroup based on the observation that IgE sensitisation to fungi was particularly prevalent in patients attending difficult to control asthma clinics [30]. Denning and colleagues proposed the term severe asthma with fungal sensitisation (SAFS) to describe this aspect of troublesome asthma and used criteria in opposition to the ABPA criteria by including an IgE of < 1000 IU/L [31, 32]. Again, SAFS means different things to different people with the oxymoron of ABPA-SAFS (ASAFS) being reported [33]. Notwithstanding the difficulty in defining severe asthma, and the questionable benefit of separating out one aspect of a condition without a strong mechanistic basis, perhaps the main weakness of the SAFS criteria is that it allows sensitisation to a range of fungi, many of which are unlikely to be involved in causing lung damage because they do not colonise the airways. We have proposed that in the present state of knowledge an inclusive approach to defining this endotype of airway disease is most appropriate and have proposed the term allergic fungal airway disease (AFAD) using the criteria of IgE sensitisation to airway colonising, thermotolerant, filamentous fungi and symptoms and signs of airway disease [34, 35]. The advocates of the exclusive approach represented by ABPA/SAFS stress the clinical relevance of these subgroups in comparison to IgE sensitisation alone, which also includes patients with mild disease. Whilst increasing numbers of studies have shown that IgE sensitisation to *A. fumigatus* not meeting the criteria for ABPA/SAFS is clinically relevant, until better biomarkers that can distinguish mild from severe disease are found it is helpful to qualify AFAD in terms of severity. The relationship between ABPA, SAFS and AFAD is illustrated in the Venn diagram in Fig. 1.

Fungal sensitisation occurs in about 3–10% of the general population [36] and 7–20% of asthmatics. The prevalence is strikingly higher in people with severe asthma, with rates between 35–75% [37]. The rate of IgE sensitisation to *A. fumigatus* was only 12% in a severe asthma population in Singapore, but was associated with severe exacerbations [38]. Fungal sensitisation is associated with life-threatening acute asthma attacks requiring ICU admissions [39] and asthma-related deaths [40]. Many fungal sensitised individuals with severe asthma do not fulfil the criteria for ABPA, so in 2006 the term SAFS was introduced [31]. However, many patients with clinically significant fungal allergy do not have severe asthma, and SAFS includes asthmatics with sensitisation to any fungus. Accumulating evidence suggests that fungi capable of growing in the lungs (thermotolerant fungi), in addition to causing an IgE mediated response, may be causing complications beyond those of their non-thermotolerant relatives [21].

The term AFAD has a liberal definition, based on the presence of IgE sensitisation to thermotolerant fungi and evidence of fungal-related lung damage [34]. As such it is more inclusive than ABPA or SAFS, being not reliant on the high total IgE measurement required for a diagnosis of ABPA yet not closely associated with the development of lung disease [29], nor restricted to severe asthmatics. Furthermore, unlike SAFS, AFAD distinguishes between sensitisation to thermotolerant and non-thermotolerant fungi.

Other terminology besides SAFS and AFAD have also been proposed in recent years. In 2018 the term Airway Mycosis was introduced as a term to reflect that, rather than innocuous colonisation, the fungi growing in airways were producing an immunologically and physiologically significant infection [41, 42]. The term encompasses both the upper and lower airways and aligns with AFAD in that it places emphasis on the presence of fungi capable of growing at body temperature in the airway driving the disease. Another term, proposed in 2020 for a paediatric population, is fungal asthma [43]. Proposed as an umbrella term to cover SAFS, ABPA/M and fungal bronchitis, it does not distinguish between thermotolerant and non-thermotolerant fungi. Fungal bronchitis is defined as bronchitis, (i.e. inflammation of the bronchi) caused by fungal infection. The condition is dominated by *Aspergillus* and *Candida* species. The symptoms are of a cough productive of discoloured, highly mucoid or even rubbery sputum (usually creamy or brown) and airway dysfunction and are often expressed as exacerbations of the underlying airway disease. Fungi are grown in the sputum and the condition responds well to antifungal agents with improvement generally noted within a month of treatment. There is often only an isolated episode, but in some people it can recur. AFAD is a risk factor, especially if *A. fumigatus* is cultured; however, the majority of people with AFAD do not get fungal bronchitis. It can, however, occur in non-sensitised individuals. Fungal bronchitis is more frequently referred to in the context of cystic fibrosis than asthma [44–46]. Recently, however, an adult retrospective analysis of predominantly individuals with asthma has suggested fungal bronchitis is a distinct clinical entity. Exacerbations were caused by a non-invasive fungal infection of the airways by thermotolerant fungi, causing impaired lung function and a chronic productive cough [47]. It should be noted, however, that fungal infection causing bronchitis is still not a widely accepted concept, although we would propose that a positive culture for *A. fumigatus* or *Candida* species in the context of an exacerbation of airway disease is a biomarker for a response to triazole antifungal therapy.

## Prevalence of AFAD

It is difficult to estimate the true prevalence of AFAD in asthma due to a lack of studies comprehensively assessing fungal sensitisation, defined as either a skin prick wheal ≥ 3 mm larger than the diluent, or a value of IgE by Immunocap assay of ≥ 0.35 IU/L towards a fungal allergen or extract. Many, including the largest study of fungal prevalence across Europe [48], have only included sensitisation to non-thermotolerant fungi such as *Alternaria alternata* and/or *Cladosporium herbarum*. Table 1 details the studies that have included both thermotolerant and non-thermotolerant fungi in their testing panel. These studies include cohorts from Europe, the USA and Asia, and test for sensitisation using between 3 and 7 fungal extracts from 12 genera, with *Alternaria* and *Aspergillus* being the two common to all 13 studies. In the paediatric studies dominated by children with mild to moderate asthma, *Alternaria* is the most common fungi causing sensitisation [49, 50]. As disease severity increases, *Aspergillus* becomes the dominant allergen [51]. In adults, *Aspergillus* is more frequently the dominating allergen, although in a study from China *Penicillium* dominated [52], and in Japan *Candida* was the most common fungal allergen detected [53, 54]. It is interesting to note that in adults, *Aspergillus* dominated European studies from countries with a temperate climate, whereas *Alternaria* dominated in Texas in the USA, which has a humid subtropical climate, and in Tokyo in Japan, another humid subtropical climate it was *Candida*. How much is attributable to geographical differences in fungal exposure and how much to variation in fungal extracts is unknown. In adults, even in predominantly non-severe cohorts, *Aspergillus* was often the most common allergen [55, 56], and those individuals sensitised to *Aspergillus* may be at higher risk of progressive lung damage than those sensitised to non-thermotolerant fungi [21]. Prevalence of fungal sensitisation varied greatly between studies, with sensitivity to more than one fungus being detected from as few as 3% of individuals in a predominantly mild to moderate asthma cohort [56] to 66% in a severe cohort [57]. Sensitivity to *Aspergillus* or *Penicillium* within those two cohorts was 2.5 and 45%, demonstrating that sensitisation to thermotolerant fungi represents a significant proportion of fungal sensitisation regardless of age or asthma severity.

**Table 1:** Prevalence of fungal sensitisation in people with asthma from cohort studies that have included, at minimum, *Aspergillus* or *Penicillium* and *Alternaria* or *Cladosporium*.

Location	No	Age, years^a^	Disease severity^b^	Sensitised to ≥ fungus, %	Sensitised to *Asp* or *Pen*, %	Commonest allergen	Fungal panel tested	Test(s) used	Study
Leicester, UK	138	5–17	61% mild–moderate, 39% severe	46	35.50	*Alt*	*Alt, Asp, Can, Clad, Pen*	SPT and sIgE	[49]
Aachen, Germany	207	1–17	25% mild, 31% moderate, 44% severe	≥ 17.2^c^	≥ 11.3^d^	*Alt*	*Alt, Asp, Clad, Pen*	sIgE	[50]
New York, NY, USA	64	2–21	50% moderate, 50% severe	39	33^d^	*Asp*	*Alt, Asp, Can, Clad, Muc, Pen, Seto*	RAST	[51]
London, UK	82	4–17	All severe	46	–	–	*Alt, Asp, Clad*	SPT and sIgE	[83]
Pirkanmaa District, Finland	485	43	Newly diagnosed adult-onset	7.40	5.4^d^	*Asp*	*Asp, Clad, Muc, Pen, Pleo, Stac*	sIgE	[55]
Norrbotten, Sweden	830	59	94% mild–moderate, 6% severe	3	2.5^d^	*Asp*	*Alt, Asp, Clad*	sIgE	[56]
Beijing, China	100	56	52% mild, 24% moderate, 24% severe	16	≥ 10 ^d^	*Pen*	*Alt, Asp, Can, Clad, Pen*	sIgE	[52]
Tokyo, Japan	160	59	25% mild, 39% moderate, 36% severe	≥ 47.5 ^c^	36.90	*Can*	*Alt, Asp, Can, Clad, Muc, Pen, Tri*	sIgE	[53]
Houston, TX, USA	307	49	15% mild, 18% moderate, 67% severe	17	≥ 8^d^	*Alt*	*Alt, Asp, Clad, Muc, Pen, Stem*	sIgE	[84]
Leicester, UK	431	51	9% mild, 91% moderate–severe	76.3^e^	59.10 ^e^	*Asp*	*Alt, Asp, Can, Clad, Pen*	SPT and sIgE	[29]
Leicester, UK	126	57	6% moderate, 94% severe	48	41	*Asp*	*Alt, Asp, Bot, Clad, Pen*	SPT and sIgE	[64]
Tokyo, Japan	124	61	All severe	29	11^d^	*Can*	*Alt, Asp, Can, Clad, Pen, Tri*	sIgE	[54]
Manchester, UK	121	49	All severe	66	≥ 45^d^	*Asp*	*Alt, Asp, Bot, Can, Clad, Pen, Tri*	SPT and sIgE	[57]

The table has been separated into paediatric and adult studies then sorted by the proportion of the cohort with severe disease

Alt: *Alternaria alternata*, Asp: *Aspergillus fumigatus*, Bot: *Botrytis cinerea*, *Can Candida albicans*, *Clad Cladosporium herbarum*, *Muc Mucor racemosus*, *Pen Penicillium chrysogenum*, Pleo: *Pleospora bjoerlingii*, Seto: *Setomelanomma holmii*, Stac: *Stachybotrys chartarum*, Stem: *Stemphylium vesicarium*, Tri: *Trichophyton rubrum*, SPT: skin prick test, sIgE: specific Immunoglobulin E

^a^Age is given as range for paediatric studies and mean for adult

^b^Severity based on either original authors description or GINA classification (1/2 mild, 3 moderate, 4–5 severe)

^c^Based on number sensitised to most common fungal allergen

^d^Based on number sensitised to most common between *Aspergillus* or *Penicillium*

^e^Cohort enriched for fungal sensitised individuals

^f^The names given here are the currently accepted names for the fungi tested. Some studies only mentioned the fungal genus; however, most named the species and the species were consistently those listed here, although some used older, now obsolete names

## Diagnosing fungal allergy

Diagnosis of fungal allergy is based on patient history and in vivo and in vitro testing. Skin prick tests (SPT) and specific serum IgE tests are commonly used to diagnose sensitisation. Whilst not as sensitive as intradermal tests [58], skin prick tests have a lower rate of false positives [59] and represent a simple diagnostic tool that can be useful for screening. The majority of SPT positive individuals are also positive by specific IgE (which is generally regarded as the reference standard), giving the SPT a high positive predictive value (95%); however, about 40% of individuals with positive IgE tests are SPT negative, which makes skin prick testing insensitive and therefore unsatisfactory as a screening tool [60]. Blood tests for specific IgE are more costly than SPTs, with the immunoassay capture (ImmunoCAP) system being the preferred platform [61]. Due to discordance between and SPT and IgE tests, some authors suggest both should be used [57]. One reason for the observed discordance may be the lack of standardisation between extracts used for SPT and IgE tests. Extracts can vary between companies, and even between batches from the same company, and many factors including fungal strain used and culture conditions can influence the allergen content and antigenicity of fungal extracts [36, 62].

There is no consensus regarding which fungi should be included in a fungal allergen panel. A balance is required between being comprehensive, and feasibility due to the nature of the tests being used, the clinical samples available (for example, the amount of blood one can obtain from a child) and cost. Based on aerobiological surveys conducted in different locations of the world and test availability, the minimum recommendation for a skin test panel was *A. alternata*, *A. fumigatus*, *C. herbarum*, *Epicoccum nigrum, Fusarium roseum* and *Penicillium chrysogenum*. This list does not include any fungi from the Basidiomycota, even though they are important in terms of exposure and many have been shown to be allergenic, because suitable extracts are not often commercially available [36]. The list also excludes species from the Mucorales, even though some are known opportunistic pathogens and allergens and ubiquitously present in the environment, and it does not include the commensals such as *Candida* or *Malassezia,* which similarly includes opportunistic pathogenic and allergenic species. In a paediatric population it has been suggested that, in a clinical setting, they can only test for three fungal allergens, *A. fumigatus*, *A. alternata* and *C. herbarum* [43]. The paediatric studies shown in Table 1 have tested between 4 and 7 fungal species, and the adult studies 3 and 7. None included *Epicoccum* or *Fusarium*. From an infectious perspective, *Epicoccum* is not thermotolerant and is not associated with human infection, therefore likely to play an aeroallergen role. *Fusarium*, however, is listed as a rare etiologic agent for ABPM [63].

**Fig. 2:**
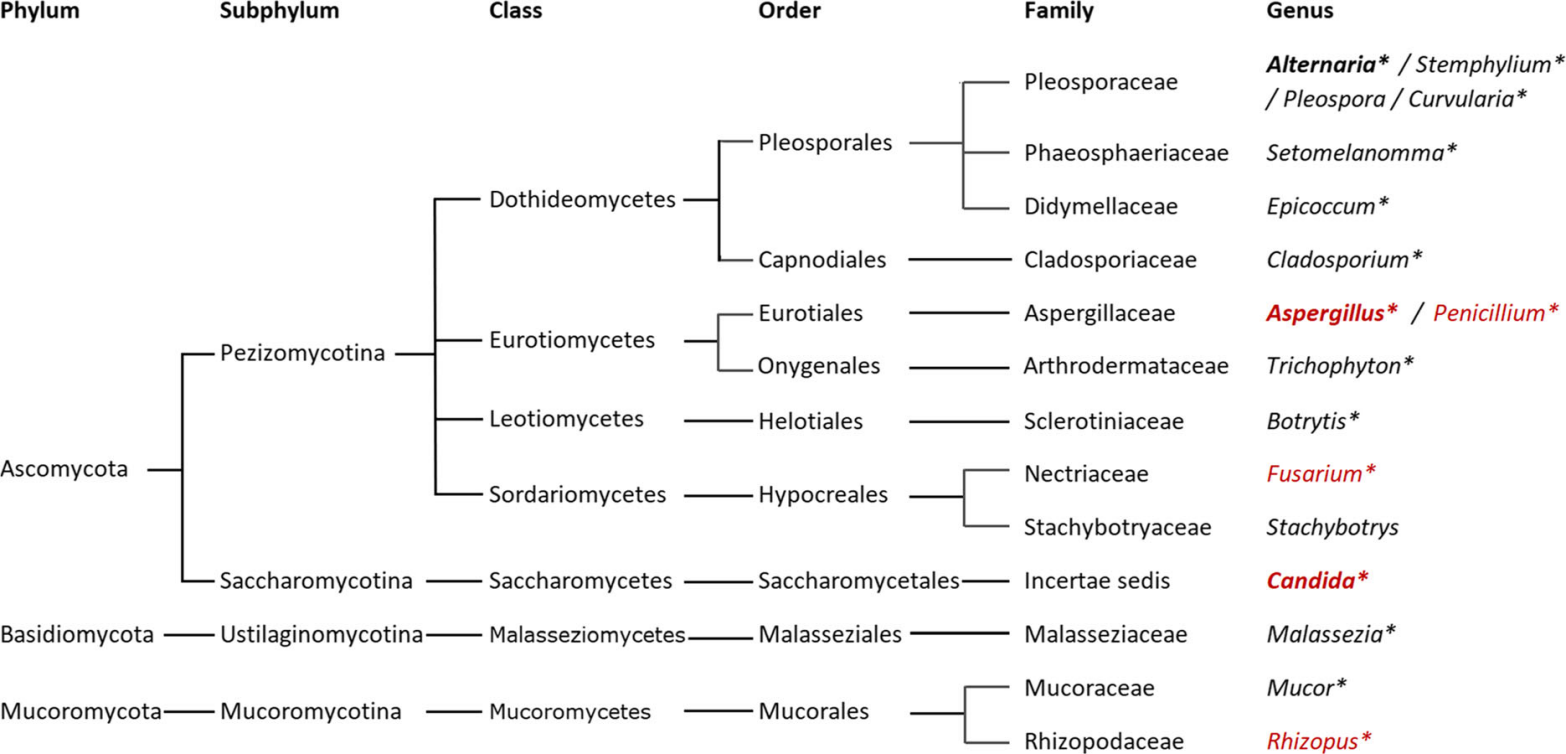
Taxonomy and phylogenetic relationships between the fungal genera mentioned in this review. Those in red are genera that include species that have been cultured at 37 °C from sputum in asthma or COPD [11, 64]. Genera in bold are our recommended fungal allergen panel if resources are limited, and those with a star have an ImmunoCap IgE assay available

Specific IgE levels in individuals sensitised to fungi often closely match the fungal phylogenetic relationships. As such, this can be taken into consideration when deciding which allergens to test [19]. Several of the fungi less commonly used in Table 1 represent very closely related fungi. *Alternaria*, *Pleospora*, and *Stemphylium* are from the same fungal family, the Pleosporaceae, and *Setomelanomma* from the same order, the Pleosporales (Fig. 2). As such, there is likely to be a high level of cross-reactivity between the allergens and testing only with *Alternaria* from that group should be sufficient. Similarly, although as a research group we do currently include *Aspergillus* and *Penicillium* [29, 49, 64, 65]; these represent two very closely related genera from the family Aspergillaceae. In our experience, sensitisation to the two fungi often co-occurs, with mono-sensitised individuals more frequently sensitised to *A. fumigatus,* suggesting *P. chrysogenum* could be excluded if resources are limited. Similarly, we often observe co-sensitisation between *Alternaria* and *Cladosporium*, both genera within the fungal class Dothideomycetes, with *Alternaria* sensitisation being the more common. It should be noted that the Dothideomycetes represents one the largest and most diverse classes of ascomycete fungi, and therefore co-membership does not infer the same level of genetic relatedness as being in the same fungal family. *Epicoccum* is also in the same order as *Alternaria.* The role for *Candida* sensitisation and colonisation in asthma is still unclear. *Candida* is an etiologic agent for ABPM [63] and fungal bronchitis in asthma [47], and antifungal treatment of fungal bronchitis when *Candida* was the causative agent has been shown to result in clinical improvement [47]. However, individuals sensitised to *Candida* in the absence of *A. fumigatus* sensitisation did not have demonstrably worse lung function or significantly more radiological abnormalities than non-fungal sensitised individuals [29]. Four of the studies in Table 1 included *Mucor*. Of the approximately 50 species within the genus, a small proportion (around six) are thermotolerant and can be opportunistic human pathogens. *Rhizopus*, another genus known to be capable of causing allergies, and a more common opportunistic pathogen than *Mucor*, is in the same fungal family (Mucoraceae) and an etiologic agent of ABPM [63]. In our limited experience only a relatively small proportion of people with asthma are sensitised to Mucorales compared to *A. fumigatus* by skin prick test, with more individuals sensitised to *Mucor* than *Rhizopus*, with the majority co-sensitised with a larger wheal to *A. fumigatus.* Lastly, occasionally specific IgE to one or more of the commercially available allergenic components of *A. fumigatus* (Asp f 1–4/6) is positive when the IgE to the extract is below the 0.35 IU/L threshold. Components should be measured if the clinical picture is suggestive of AFAD, but the specific IgE is negative.

## Identification of fungi from respiratory samples

To determine if a fungus is having an effect beyond sensitisation, it is important to be able to detect the fungus in the airways. Early studies were hampered by an inability to easily and reproducibly culture fungi from respiratory tract samples, and there is often a misconception that, because most clinically relevant fungi have spores that are ubiquitous, a positive culture is not clinically meaningful [41]. Most countries including the USA, Canada and Australia have no national standard guidelines for processing respiratory samples. The UK is an exception, however, the original method [66], still used in many clinical microbiological laboratories, was shown to be highly insensitive at culturing fungi from sputum [67, 68]. More recently, a modified protocol has been published recommending a higher volume approach [69].

Using a high volume culture approach to detect *A. fumigatus* in patients with moderate to severe asthma who were IgE sensitised to *A. fumigatus*, more than 60% were found to be culture positive compared to less than 10% historically using the UK standard approach [70]. *A. fumigatus* was isolated from around a third of those sensitised to only *A. fumigatus*-IgG or non-sensitised and from only 7% of healthy control subjects. Furthermore, this study found that the *A. fumigatus*-IgE sensitised group had worse lung function than those who were non-sensitised and had more bronchiectasis [70]. This finding was subsequently confirmed in a different population of severe asthmatics [71]. High rates of *A. fumigatus* culture from sputum were subsequently found in other fungal sensitised severe asthma cohorts [72].

*A. fumigatus,* whilst the most common, is not the only thermotolerant fungus cultured from sputum. Using the high volume approach 27 different filamentous fungi were obtained [64], predominantly species of *Aspergillus* and *Penicillium*. Lung function was found to be significantly lower in those with a positive fungal culture compared to those who were culture-negative. Indeed individuals who were both fungal culture positive and fungal sensitised had a 22% drop in FEV_1_ (% predicted) compared to the group who were non-fungal sensitised and culture negative. Interestingly, sensitisation to *Aspergillus* and *Penicillium* was significantly higher in the culture positive group whilst there was no significant difference between the culture positive and culture negative groups with regard to sensitisation to the typical fungal aeroallergens *Alternaria*, *Cladosporium* or *Botrytis*, which are not normally associated with thermotolerance [64].

Given the inherent problems perceived with fungal culture, there is growing interest in utilising molecular DNA based techniques, primarily quantitative polymerase chain reaction (qPCR)-based methods, as an alternative to, or complement for culture as a way of identifying fungi from respiratory samples. Many of the approaches for detecting fungi directly from respiratory tract specimens, including the use of panfungal PCR assays, multiplex of pathogen-directed assays, and technologies using real-time PCR, isothermal methods and probe-based assays were reviewed recently in the context of cystic fibrosis [73], but apply equally to asthma. One of the advantages of molecular techniques may also be a detriment in that they can be too sensitive; hence, determining a clinically relevant threshold will become very important. A recent study investigating the airway mycobiome in asthma and health found a remarkable similarity between the major fungi detected, with *A. fumigatus* and *Candida albicans* being highly prevalent and abundant across all subjects regardless of asthma status, disease severity or fungal sensitisation status [65]. The main differences detected were shifts in the balance of fungi associated with asthma status, asthma duration and biomarkers of inflammation, with members of the *Aspergillus niger* and *Cryptococcus humicola* species complexes highlighted as potentially playing unexpected roles in the pathogenesis of asthma. A similar mycobiome study comparing samples from people with bronchopulmonary aspergillosis, SAFS, asthma and healthy controls found the *A. fumigatus* complex to be the most common fungus detected in all individuals, regardless of disease, and it was the use of qPCR that enabled them to determine that corticosteroid treatment was significantly associated with fungal load [74]. The airway mycobiome consists of thermotolerant fungi, likely colonising the airways, and non-thermotolerant species highly abundant from air samples obtained on the same day as the clinical samples [65]. Analysis of the mycobiome in a large cohort of patients with COPD revealed a cluster with a high rate of exacerbations and poor outcome linked to the presence of *Aspergillus*, *Penicillium* and *Curvularia* and IgE sensitisation to these fungal genera [75].

Consistent with pollen allergy that is most commonly caused by pollen from wind-pollinated plants that dominate the pollen air spora, fungal sensitisation tends to be against commonly encountered fungal species. Some such as species from the genera *Candida*, *Malassezia,* and *Trichophyton* are human commensals or dermatophytes, but the majority have environmental sources and dominate the fungal air spora [19, 36]. In many areas, including the UK and USA, outdoor airborne fungal spores exceed pollen concentrations by 100–1000-fold [36, 76] and recently have been shown to exceed bacterial concentrations [77]. Fungi are also commonly found indoors, even in non-mould-complaint homes [78] and exposure to *A. fumigatus* indoors has been associated with fungal colonisation in asthma [79]. In COPD, exposure and sensitisation to a number of common indoor and outdoor fungal species were related to sub-optimal outcomes [80], and warrants a comparable study in asthma.

## Pathophysiological abnormalities related to fungal allergy

To understand the role of fungi in lung disease we need to determine the clinically important pathophysiological abnormalities that are related to fungal allergy [21]. A large cohort (*n* = 431) of asthmatics enriched for IgE sensitisation to fungi were recruited in a cross-sectional study to determine the relationship between immunological biomarkers of fungal allergy and evidence of lung damage in asthma [29]. Around three quarters of subjects were sensitised to one or more fungi. Whilst being well matched for age, gender, smoking status and GINA score, the fungal sensitised group were more likely to have early-onset atopic asthma than the non-sensitised group. Subjects sensitised to thermotolerant filamentous fungi (*Aspergillus* and *Penicillium*) were found to have lower lung function than those sensitised to thermotolerant yeasts (*Candida*, 73% predicted versus 77% predicted) and significantly lower lung function than those sensitised to non-thermotolerant fungi (*Alternaria* and *Cladosporium*, 73% predicted versus 85% predicted, *p* < 0.05), or not fungal sensitised (73% predicted versus 82% predicted, *p* < 0.001). Furthermore, IgE sensitisation to *A. fumigatus,* independent of atopic status (IgE sensitised to house dust mite, dog, cat, grass or tree pollen), was associated with a significantly lower post bronchodilator FEV_1_ compared to those who were just atopic (72% predicted versus 84% predicted, *p* < 0.005).

Bronchiectasis, tree-in-bud appearances and the presence of collapse/consolidation were significantly more frequent in individuals sensitised to fungi (*p* < 0.05), particularly in the group sensitised to thermotolerant fungi. *A. fumigatus* IgE sensitisation was significantly associated with five radiological abnormalities; bronchiectasis (*p* < 0.001), tree-in-bud (*p* < 0.001), fleeting shadows (*p* < 0.001), collapse/consolidation (*p* < 0.002) and fibrosis (*p* < 0.05). In contrast, *A. fumigatus* IgG sensitisation was only significantly associated with bronchiectasis (*p* < 0.005), tree-in-bud (*p* < 0.05) and fleeting shadows (*p* < 0.05), and at lower significance than for IgE, whilst total IgE was only significantly associated with tree-in-bud (*p* < 0.01) and fleeting shadows (*p* < 0.001). A higher prevalence of radiological abnormalities was seen in those sensitised to the thermotolerant fungi whereas sensitisation to the thermotolerant yeasts or the non-thermotolerant fungi was not associated with any radiological abnormalities [29]. Taken together these data demonstrated that the association with fungal sensitisation and fixed airflow obstruction is limited to the thermotolerant filamentous fungi and is not simply a function of atopy. Furthermore, whilst total IgE was associated with fleeting shadows (nowadays a rarely seen feature) and to a lesser extent tree-in-bud, it was not associated with fixed airflow obstruction or any other radiological abnormality.

## Management of AFAD

To a large extent management of AFAD is similar to the management of the underlying airway disease, and we would advocate an approach based on deconstructing the various pathophysiological abnormalities into their component parts [9]. As an eosinophilic pattern of disease inhaled corticosteroids are a keystone of therapy to help control exacerbations. Whilst there is a theoretical risk of encouraging fungal colonisation, in practice this does not seem to be a problem although the minimum dose to achieve control should be used. In more severe cases, as is the case with eosinophilic asthma without AFAD, low-dose continuous oral corticosteroids are necessary to achieve control. Increasingly, systemic corticosteroids are being supplanted by anti-T2 biological therapy such as mepolizumab and benralizumab. Whilst there are no prospective controlled trials of these drugs in AFAD subjects in a post-trial analysis of patients with AFAD given mepolizumab the reduction in exacerbations was as great, if not greater than, subjects who were not sensitised to *A. fumigatus* [81]. A key pathological component of AFAD is obstruction of the bronchi with viscid mucus, which causes persistent impaction. This is a feature of severe eosinophil airway disease in general although the mucus in AFAD appears particularly sticky and impacting as seen in the unusual but archetypal presentation of lobar collapse. There are no specific therapies for this at the moment, with therapy aimed at reducing eosinophilic inflammation. As noted above the common characteristic features of AFAD are those of lung damage, with fixed airflow obstruction, bronchiectasis and lung fibrosis prominent. Fixed airflow obstruction leads to symptoms of chronic breathlessness mimicking to a degree smoking related airflow obstruction (COPD) which AFAD can also complicate. Whilst bronchodilators have a place in therapy there are no specific treatments, although pulmonary rehabilitation is of value. Bronchiectasis can lead to episodes of bacterial and fungal bronchitis. The former require broad-spectrum antibiotics guided by sensitivities of the offending bacterial species together with regular physiotherapy. Fungal bronchitis is not a term that is widely used in the scientific literature, or in common clinical parlance, but seems to us an apposite term to describe bronchitis (inflammation of the bronchi) caused by fungal infection which is usually due to *Aspergillus* or *Candida* species. The symptoms are of a cough, productive or discoloured sputum which is mucoid or even rubbery in consistency, often with a creamy or brown colour, and airway dysfunction. It often presents in the context of exacerbations of airway disease that are unresponsive to systemic corticosteroids and broad-spectrum antibiotics. Sputum culture is positive for the offending fungal species and it is generally responsive to triazole antibiotics with improvement seen within a month of the start of treatment [47]. Whilst it is a feature of AFAD, particularly when *A. fumigatus* is the fungal culprit, it can occur in subjects without fungal allergy and also without bronchiectasis. It is not uncommon, but the insensitive approaches to culture and scepticism amongst microbiologists regarding the clinical significance of a positive sputum fungal culture, particularly for growth of *Candida albicans*, results in it being under recognised. It can also be difficult to treat because of issues of cost, adverse effects of antifungal therapy, poor absorption and azole resistance. The place of antifungal therapy in AFAD remains uncertain. Whilst open studies have often reported a benefit, placebo controlled, blinded studies have shown either no benefit or a modest improvement at best compared to standard of care, which these days probably includes biological therapy. Clinical practice, which our experience supports, would suggest that in the majority of patients with AFAD the benefits of azole therapy are not outweighed by the downsides. However, where fungal bronchitis is present, particularly in the context of difficult to treat exacerbations, they are an important adjunct to therapy and can lead to a dramatic improvement in symptoms in relatively short order. We would propose that a positive sputum fungal culture is a useful biomarker of a response to antifungal therapy even in the case of *Candida* species if it is persistent.

## Summary

The term AFAD was conceived with a liberal definition based on IgE sensitisation to thermotolerant fungi and evidence of fungal-related lung damage. AFAD represents a continuous spectrum of disease severity at which ABPA/ABPM are the extreme. It is more inclusive than ABPA or SAFS, being not reliant on high total IgE or restricted to severe asthma. However, unlike SAFS, it distinguishes between thermotolerant fungi capable of causing infection and sensitisation, and non-thermotolerant fungi that still act as important allergens. Sensitisation to thermotolerant filamentous fungi (*Aspergillus* and *Penicillium*), but not non-thermotolerant fungi (*Alternaria* and *Cladosporium*) is associated with lower lung function and radiological abnormalities (bronchiectasis, tree-in-bud, fleeting shadows, collapse/consolidation and fibrosis). For antifungals to play a role in treatment, the focus should be on fungi capable of growing in the airways thereby causing a persistent chronic allergenic stimulus and releasing tissue damaging proteases and other enzymes which may disrupt the airway epithelial barrier and cause mucosal damage and airway remodelling [43]. Testing for the presence of fungi in the airways and for sensitisation to fungi are clearly important in understanding the role of AFAD in asthma; however, the methodology used for both requires standardisation [82] and a consensus as to which fungi to test for would be beneficial. Nevertheless, all patients with IgE sensitisation to thermotolerant fungi in the context of asthma and other airway disease are at risk of progressive lung damage, and as such should be monitored closely irrespective of a diagnosis of ABPM.
